# Patients’ preferences within randomised trials: systematic review and patient level meta-analysis

**DOI:** 10.1136/bmj.a1864

**Published:** 2008-10-31

**Authors:** Preference Collaborative Review Group

## Abstract

**Objective** To systematically review fully randomised patient preference trials and to explore the impact of preferences on attrition and outcome by meta-analysis of patient level data.

**Data sources** Citation search using Science Citation Index and Google Scholar and search of the main electronic databases (Medline, CINAHL, Embase, and AMED) with a combination of key words.

**Study selection** Fully randomised patient preference trials that compared treatments for any clinical condition were included. Other types of preference trials and crossover trials were excluded. Other inclusion criteria: participants aged 16 years and over; primary, self-reported outcomes measured on a continuous numerical scale. From 167 studies identified and screened, 17 were identified as fully randomised patient preference trials.

**Data synthesis** Of the 17 trials identified, 11 authors provided raw data for the meta-analysis. Data collected were baseline and follow-up data for the main outcome, randomised allocation data, preference data, and demographic data. Baseline and first post-intervention follow-up data for the main outcome were standardised. To improve homogeneity, data for only the eight musculoskeletal trials (n=1594) were combined. To estimate the effects of preferences on outcomes and attrition, three groups were compared: patients who had a preference and were randomly allocated to their preferred treatment; patients who had a preference and were randomly allocated to the treatment they did not prefer; and patients who had no preference.

**Results** Patients who were randomised to their preferred treatment had a standardised effect size greater than that of those who were indifferent to the treatment assignment (effect size 0.162, 95% confidence interval 0.011 to 0.314; P=0.04). Participants who received their preferred treatment also did better than participants who did not receive their preferred treatment (effect size 0.152, −0.035 to 0.339), although this was not statistically significant (P=0.11). Participants allocated to their undesired treatment had outcomes that were no different from those who were indifferent. Participants who were allocated to their undesired treatment were less likely to be lost to first follow-up compared with indifferent participants (odds ratio 1.70, 1.076 to 2.693; P=0.02). No difference was found in attrition between patients allocated to their preference and those who were indifferent.

**Conclusions** Preferences among patients in musculoskeletal trials are associated with treatment effects. In open randomised trials, preferences should be ascertained before randomisation.

## Introduction

The randomised controlled trial is acknowledged as the most scientifically rigorous study design for evaluating medical interventions.^1^ Although random allocation is intended to evenly distribute characteristics of participants that may affect outcome and remove selection bias, it may not deal with other potential biases. One of these is patients’ preferences. Random allocation will distribute patients with a preference for a given intervention between the intervention groups in similar proportions, as it does with physical characteristics such as weight or height. What it cannot do, however, is deal with the post-randomisation effects of these preferences on treatment outcomes. Because patients’ preferences are not dealt with in the randomisation process, they are viewed as a potential threat to the validity of trials.^2^ The effect of patients’ preferences on treatment outcomes in randomised controlled trials is, however, uncertain.^3^

Patients with strong preferences may decline to participate; in a trial in which strong preferences exist and a large number of patients refuse randomisation, the external validity will be adversely affected. When this occurs, generalisability of the results to a wider population will be limited.^4^ If patients with preferences consent to randomisation, this may also affect its internal validity.

In a randomised controlled trial, patients may have a preference either for the standard treatment or for the new treatment being evaluated or may be indifferent to both treatments. If patients with preferences consent to be randomised then some patients will get their preferred treatment and others will not. Those who receive their preferred treatment might be better motivated and comply better with the treatment programmes and report better outcomes.^5^ Patients who do not receive their preferred treatment may experience “resentful demoralisation,”^6^ may be less motivated, may not comply with the treatment programme, may not report accurately during follow-up, and may even drop out of the trial and thereby introduce bias that affects the internal validity of the trial.^5^ Effects of preference are likely to be more apparent in unblinded trials in which patients are aware of the treatment they are receiving and the outcome measure is subjective and self reported by the patient.

As well as the direct effects of patients’ preferences on compliance and motivation, a “therapeutic effect” of patients’ preferences can occur.^7^ These are the psychological effects that influence outcomes and are similar to the placebo effect.^7^ The therapeutic effects of patients’ preferences are problematic for trials because these seek to determine or assess the physiological or pharmacological effects of an intervention. Disentangling the psychological effects of patients’ preferences from the physiological effects is complex.^8^

One approach to dealing with patients’ preferences is the partially randomised preference design also known as a patient preference trial or comprehensive cohort design.^2^ ^5^ Patients without strong preferences for a treatment are randomised, and those with strong preferences are given their choice, resulting in a four armed trial.^2^ Such a design enables comparisons between patients with and without a preference and an exploration of patients’ characteristics associated with preference. An important disadvantage of this design is that the outcome may be affected by uncontrolled confounders in the non-randomised groups,^9^ which may bias the results.^10^ Whether the randomised group should be compared with the non-randomised group is therefore debatable.^9^ ^6^

An alternative approach is a standard randomised controlled trial in which, after participants have given consent in the usual way and before randomisation, patients’ preferences are recorded: this design would be a fully randomised preference trial.^6^ These preferences are then taken into account in the analysis of the trial. Such an approach has been used in some trials,^w1^ and at least one apparently observed a preference-treatment interaction.^w2^ This interaction, however, was not statistically significant. A potential criticism of this approach is that ignoring patients’ preferences and proceeding with randomisation is unethical. However, a counter argument is that ignoring preferences may be ethical if all the treatments being offered are believed to be effective and patients give fully informed consent.^w3^

Detecting patients’ preference-treatment interactions within trials is difficult, as trials are not sufficiently powered to detect them.^10^ ^4^ However, Torgerson and Sibbald suggested standardising the way in which information on preferences is obtained in trials so that the trials could be used in meta-analysis to assess the effect of preferences on outcomes.^11^

King and colleagues recently systematically reviewed the effects of participants’ preferences in randomised controlled trials.^5^ ^12^ They investigated the effects of preferences on outcomes and recruitment to trials and the influence of preferences on attrition. Their review identified 34 randomised controlled trials, but only two of these were fully randomised patient preference trials.^13^ ^w1^ The review, which used evidence from partially randomised preference trials, found no evidence that preferences influenced attrition. It found some evidence of an effect on outcomes in some of the preference trials and no effect on outcomes in the two conventional randomised controlled trials. The trials and studies included in the review were very heterogeneous, and the authors reported that they were therefore unable to “reach definitive conclusions for any particular clinical field.”^5^

Here we report the results of a systematic review and an individual patient data analysis of fully randomised patient preference trials. The aim of the review was to assess whether preferences had an interaction with effectiveness of treatment and whether attrition from the study was different between patients who received their preferred treatment, those who did not, and those who had no preference. This review differs from the previous review by King and colleagues in that we were interested only in fully randomised preference designs and we also sought patient level data for our analysis.

## Methods

We wanted to identify all trials that have used the fully randomised preference trial design. The extensive systematic review by King and colleagues (2005) searched for papers published between 1966 and September 2004 and identified only two that were fully randomised patient preference trials.^5^ To find additional papers we did a citation search using Torgerson et al (1996) and Torgerson and Sibbald (1998),^6^ ^11^ which were the first papers to describe the fully randomised patient preference trial. We used Science Citation Index and Google Scholar to do the citation search. In Science Citation Index, we selected the two papers on which the citation search was to be based. The database then identified all articles that had cited the papers. When we used Advanced Scholar Search in Google Scholar, we selected the subject areas “Biology, life sciences, and environmental science” and “Medicine, pharmacology, and veterinary science,” and the date range 2000 to October 2006. We did our first search on the terms “random, trial, preference, Torgerson, Sibbald” and a second search on the terms “random, trial, preference, Torgerson, Klaber”. We also made searches in Medline, CINAHL, Embase 1996 to 2006, and AMED on the search terms “patient preference$ trial$”. We identified relevant studies initially by title and abstract and finally by full text. In addition, we included trials that were known to us through personal knowledge.

We included only fully randomised preference trials.^6^ Other inclusion criteria were participants 16 years and over and primary self reported outcomes measured on a continuous scale. We included only trials with self reported outcomes, as trials with outcomes reported by clinicians (or another assessor) are unlikely to be influenced by patients’ preferences. Additionally, we chose to focus on only one self reported primary outcome and exclude secondary outcome measures to avoid type I error. If trialists had used more than one primary outcome measure, we used the measure that was most common to all the studies, as this would tend to ameliorate the statistical heterogeneity between the studies. We selected outcomes measured on a numerical scale, as these would be more powerful than dichotomous outcomes. We excluded trials in which patients were given their choice of treatment and crossover trials. We checked the generation of the randomisation sequence, concealment of randomisation allocation, and systematic differences in attrition of participants between the comparison groups to ensure the methodological quality of the included studies.

We initially approached the authors of the identified trials by email to ask if they would be willing to release their data for the study. If they expressed an interest, we sent them the protocol for this study with a request for the raw data. Because the studies used different outcome measures, we calculated standardised baseline and outcome scores for all patients by dividing their scores by the baseline standard deviation for the sample population for that trial. In addition, most trials had multiple follow-up points. We therefore decided to use the first follow-up point after treatment had finished, as this would tend to minimise loss of data and be most likely to show an effect of preference if one existed.

### Analysis

To analyse if patients’ preferences influence outcomes, we used multiple regression with dummy variables for categorical predictors. We used SPSS version 14. The dependent variable was the standardised first follow-up score, and the predicting variable was patient’s preference for treatment. We adjusted the analysis for baseline scores and categorical variables of trial and treatment allocation, which adjusted for treatment effects of individual interventions. In the regression analyses, we treated study as a fixed effect. We checked this assumption with a heterogeneity test and found no evidence of heterogeneity. For calculating the odds ratios for response to first follow-up questionnaires, we used logistic regression analysis. The dependent variable was missing data at first follow-up, and in the analysis we adjusted for age, sex, trial, and severity of condition at baseline. We compared the outcomes of three preference groups: patients with a preference who got what they wanted, those with a preference who did not get what they wanted, and those who had no preference (that is, they were indifferent). We first compared the two preference groups with the indifferent group; then we compared patients with a preference who were randomised to their desired treatment with those who were randomised to their undesired treatment and the indifferent group. We assessed the methodological quality of the trials and found them to be adequate, so we did not need to include this in the analysis.

## Results

Our search identified 167 possibly relevant studies (figure[Fig fig1]). After screening the abstracts and full papers, the searches identified 17 trials that met the inclusion criteria of this study (table 1), and we were able to obtain the patient level data for 11 studies (table 2).^w1-w12^ All the trialists had used simple preferences, whereby the participants indicated which treatment they preferred or if they were indifferent to the treatments. In eight of the 11 studies, the questionnaires had been completed “alone” (that is, not in the presence of a healthcare professional or researcher) by the participants. A researcher was present in the trial by Johnson and colleagues (2007) but was advised not to help the participants to complete the questionnaires.^w7^ The information the participants had available to them on the treatments was provided in information sheets. In the trial by Hay and colleagues (2003) and Thomas and colleagues (2004),^w5 w6^ patients were asked to complete the baseline questionnaires before the visit of a research nurse who was blind to treatment allocation, and patients did not complete follow-up questionnaires in the presence of any treating healthcare professionals. In the trial by Adamson and colleagues (2005),^w3^ patients were asked what their treatment preference was at the feedback session immediately before allocation of treatment; a standard description was used for the two treatments, and the trialists advised that they were scrupulous in establishing a sense of equipoise in the minds of all personnel involved in the trial.

**Figure fig1:**
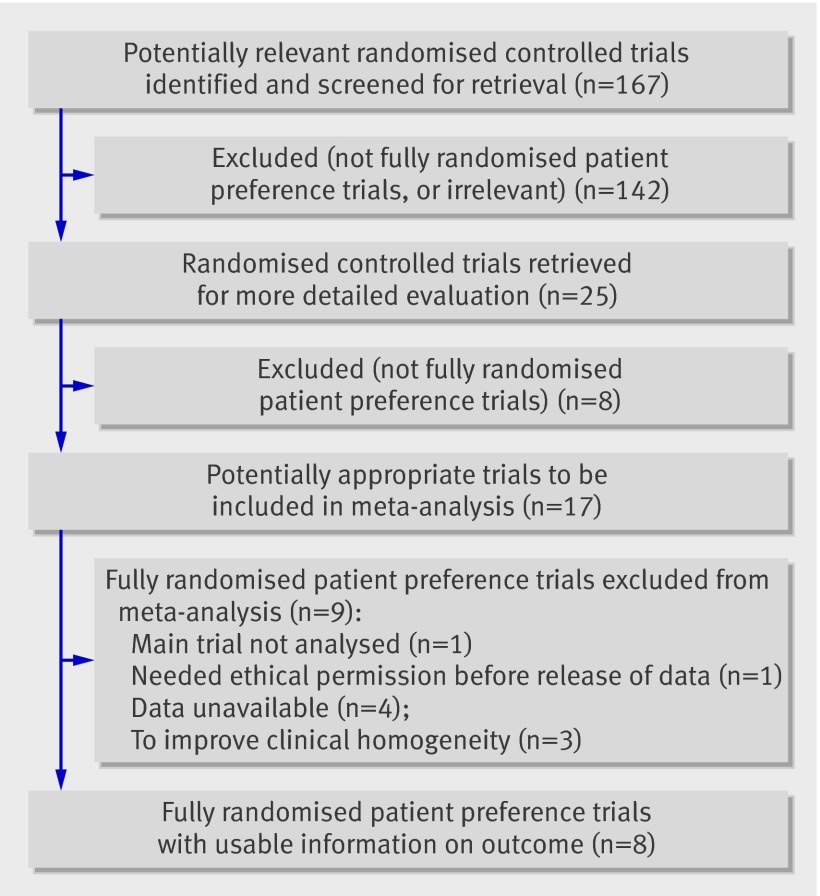
Flow chart of studies included in meta-analysis

**Table 1 tbl1:** Description of identified trials

Adamson et al, 2005^w3^	Therapy preference and treatment outcome in clients with mild to moderate alcohol dependence	Science Citation Index and known to York Trials Unit	Yes
Carr et al, 2005^w4^	A randomized trial comparing a group exercise programme for back pain patients with individual physiotherapy in a severely deprived area	DT was joint investigator	Yes
Hardy et al*, 1995^13^; Shapiro et al, 1994^16^	Effects of treatment duration and severity of depression on the effectiveness of cognitive-behavioural and psychodynamic interpersonal psychotherapy	Cited in systematic review by King et al, 2005	No
Hay et al, 2003^w5^; Thomas et al, 2004^w6^	What influences participants’ treatment preference and can it influence outcome? Results from a primary care-based randomised trial for shoulder pain	Science Citation Index	Yes
Johnson et al, 2007^w7^	Active exercise, education and cognitive behavioural therapy for persistent disabling low back pain: a randomised controlled trial	DT was co-author on trial (publication pending at the time of review)	Yes
Kendrick et al, 2005^17^	A trial of problem-solving by community mental health nurses for anxiety, depression and life difficulties among general practice patients: the CPN-GP study.	Science Citation Index	No
Kitchener et al, 2006^w8^	Laparoscopic versus open colpo-suspension—results of a prospective randomised controlled trial	DT was joint investigator	Yes
Klaber Moffett et al, 1999^w1^	Randomised controlled trial of exercise for low back pain: clinical outcomes, costs, and preferences	Cited in systematic review by King et al, 2005. Science Citation Index, and DT was co-investigator	Yes
Klaber Moffett et al, 2005^w2^	Randomised trial of a brief physiotherapy intervention compared with usual physiotherapy for neck pain patients: outcomes and patients’ preference	Science Citation Index, and DT was co-investigator	Yes
Klaber Moffett et al, 2006^w9^	Randomized trial of two physiotherapy interventions for primary care neck and back pain patients: “McKenzie” vs brief physiotherapy pain management	Science Citation Index, and DT was co-investigator	Yes
McLean et al, publication pending^w10^	A randomised controlled trial comparing graded exercise treatment and usual physiotherapy for patients with neck pain	Found from contacting JKM (publication pending at the time of review)	Yes
Pepper et al, in press^18^	Female urinary incontinence: women’s preference for group or individual treatment	DT saw conference poster (publication pending at the time of review)	No
Salisbury et al, 2005^w11^	Evaluation of a general practitioner with special interest service for dermatology: randomised controlled trial.	DT noted when reading journal	Yes
Salter et al, 2006^w12^	Acupuncture for chronic neck pain: a pilot for a randomised controlled trial.	DT advised on trial design (publication pending at the time of review)	Yes
Sherman et al, 2005^19^	Comparing yoga, exercise, and a self-care book for chronic low back pain. a randomized controlled trial	DT noted when reading journal	No
Simpson et al, 2000^20^	A randomised controlled trial to evaluate the effectiveness and cost-effectiveness of counselling patients with chronic depression	Cited in article by van Schaik et al, 2004, which was found on Medline	No
Unützer et al, 2002^21^	Collaborative care management of late-life depression in the primary care setting	Cited in article by van Schaik et al, 2004, which was found on Medline	No

*Reported strengths of preferences for interventions but not proportions.

**Table 2 tbl2:** Summary of studies included in systematic review

Authors	Population	Sample size (No)	Mean age (years)	Intervention and comparison groups	Primary outcome(s)	Follow-up points for primary outcome
Adamson et al, 2005^w3^	Patients with mild to moderate alcohol dependence	124	35.7	Two types of counselling: four sessions of motivational enhancement therapy and non-directive reflective learning, and no further counselling	Drinking; global functioning (global assessment scale)	6 months
Carr et al, 2005^w4^	Patients with non-specific low back pain	237	42.2	Group exercise programme (“back to fitness”) *v* individual physiotherapy	Roland Morris disability questionnaire	3 months*, 12 months
Hay et al, 2003^w5^; Thomas et al, 2004^w6^	Shoulder pain	207	57.4	Steroid injection *v* physiotherapy	Shoulder disability questionnaire	6 weeks*, 6 months
Johnson et al, 2007^w7^	Low back pain	234	47.9	Educational pack containing booklet, audio cassette with advice on self management *v* aforementioned plus group programme of exercise and education using a cognitive behavioural therapy approach	Roland Morris disability questionnaire	3*, 9, and 15 months
Kitchener et al, 2006^w8^	Women with proved stress urinary incontinence requiring surgery	291	50.6	Laparoscopic *v* open colposuspension surgery	Satisfaction with outcome (Bristol female lower urinary tract symptom questionnaire)	6 weeks and 6, 12 and 24 months
Klaber-Moffett et al, 1999^w1^	Low back pain	187	41.9	Progressive exercise programme *v* usual care (under care of doctor and in some cases referred for physiotherapy)	Roland Morris disability questionnaire	6 weeks*, 6 months, and 12 months
Klaber Moffett et al, 2005^w2^	Patients with sub-acute and chronic neck pain	268	48.0	Brief physiotherapy intervention *v* “usual” physiotherapy	Northwick Park neck pain questionnaire	3 months*, 12 months
Klaber Moffett et al, 2006^w9^	Back and neck pain	315	45.0	Cognitive behavioural principles (solution finding approach) *v* physical therapy (McKenzie approach)	Roland Morris disability questionnaire for back pain patients; Northwick Park neck pain questionnaire for neck pain patients.	6 weeks*, 6 months, and 12 months
McLean et al, publication pending^w10^	Neck pain	151	54.0	Graded exercise treatment *v* usual physiotherapy	Northwick Park neck pain questionnaire	6 weeks, 6 months*, and 12 months
Salisbury et al, 2005^w11^	Non-urgent skin problems	556	46.2	Management by general practitioner with special interest *v* usual hospital outpatient care	Dermatology life quality index	6 weeks, 9 months
Salter et al, 2006^w12^	Patients with chronic neck pain	24	56.5	Acupuncture and usual general practitioner care *v* usual general practitioner care	Northwick Park neck pain questionnaire	1 month, 3 months*

*Follow-up data used in meta-analysis (first follow-up data after intervention selected).

The proportion of patients who had a preference in the 11 trials ranged from 16% for a trial of a solution finding approach to back pain to 85% for a trial comparing general practitioner’s care with hospital care for non-urgent skin problems (table 3).^w9 w11^ The median preference rate was 56% (interquartile range 43-63%). The data show, therefore, that for this sample of fully randomised preference trials, most trial participants were not indifferent to the treatment to which they were going to be allocated.

**Table 3 tbl3:** Prevalence of preferences and characteristics of patients in trials. Values are numbers (percentages) unless stated otherwise

Authors	Sample size	Female	Mean (SD) age (years)	Indifferent	With preference	With preference for “experimental” treatment	With preference for control: usual care or alternative treatment	Missing preference data
Adamson et al, 2005^w3^	122*	52 (42)	35.7 (35.7)	16 (13)	102 (84)	90 (88)	12 (12)	4 (3.3)
Carr et al, 2005^w4^†	237	133 (56)	42.2 (10.9)	152 (64)	70 (30)	10 (14)	60 (86)‡	15 (6)
Hay et al, 2003^w5^; Thomas et al, 2004^w6^†	207	110 (53)	57.4 (13.4)	81 (39.1)	125 (60.4)	83 (66)	42 (34)‡	1 (0.5)
Johnson et al, 2007^w7^†	234	140 (60)	47.9 (11.2)	100 (42.7)	134 (57.3)	114 (85)	20 (15)	0 (0)
Kitchener et al, 2006^w8^	291	291 (100)	50.3 (10.5)	141 (48.5)	144 (49.5)	134 (93)	10 (7)	6 (2.1)
Klaber Moffett et al, 1999^w1^†	187	106 (57)	41.9 (8.9)	69 (36.9)	118 (63.1)	118 (100)	(0)	0 (0)
Klaber Moffett et al, 2005^w2^†	268	168 (63)	48.3 (14.6)	142 (53)	126 (47)	43 (34)	83 (66)‡	0 (0)
Klaber Moffett et al, 2006^w9^†	315	188 (60)	45.0 (15.1)	261 (82.9)	51 (16.2)	37 (73)	14 (27)	3 (1.0)
McLean et al, publication pending^w10^†	151	90 (60)	54.0 (14.3)	66 (43.7)	82 (54.3)§	34 (45)	41 (55)‡	3 (2.0)
Salisbury et al, 2005^w11^	556	327 (59)	48.0 (19.1)	63 (11.3)	474 (85.3)	328 (69)	146 (31)	19 (3.4)
Salter et al, 2006^w12^†	24	18 (75)	47.7 (16.5)	9 (37.5)	15 (62.5)	15 (100)	(0)	0 (0)

*Two missing people in dataset.

†Trials included in patient level data meta-analysis.

‡Physiotherapy was control treatment in these trials.

§Seven participants removed from dataset for meta-analysis as they had preference for treatment not available in trial.

Of the 11 studies with patient level data, eight (n=1594) were interventions for musculoskeletal conditions. Because the data we obtained were dominated by musculoskeletal studies whose reported outcomes were very much in accordance (that is, they reported outcomes on pain and disability), we decided to restrict our pooled analysis to these studies. Consequently, clinical heterogeneity would be reduced. We found no evidence of statistical heterogeneity between studies (χ^2^=6.02, df=8; P=0.64).

Within the musculoskeletal trials, we examined whether patients with a preference were statistically significantly different in terms of age, sex, and standardised baseline scores from participants who were indifferent. We found no differences in standardised baseline scores (mean 0.261 for preference group; 0.267 for indifferent group; 95% confidence interval for mean difference −0.068 to 0.177; P=0.38) but a difference in age (mean 48.57 for preference group; 46.99 for indifferent groups; 0.21 to 2.96; P=0.024). Women were more likely to have a preference, although this was not significantly different (46.3% (n=439) for women; 42.3% (n=271) for men; −1% to 9%; P=0.12).

After adjustment for baseline score, trial, and treatment allocation, in terms of treatment effect overall, patients who received their preference had significantly greater improvements than did those who were indifferent (mean effect size 0.162, 95% confidence interval 0.011 to 0.314; P=0.036). In contrast, contrary to expectations, those patients with a preference who did not receive their preferred treatment had only a slight, non-significant difference favouring an improvement relative to the indifferent group (effect size 0.011, −0.142 to 0.164; P=0.89). When we compared patients randomised to their preferred treatment with those randomised to their unpreferred treatment, we found an effect size of 0.152 (−0.035 to 0.339), which was not statistically significant (P=0.11). The total number of participants included in the analysis to compare the effects of preferences on outcomes was 1398 (table 4).

**Table 4 tbl4:** Number (percentage) of participants included in analysis to estimate effect of patients’ preferences on outcomes

Preferences	Frequency
Did not receive preference	331 (23.7)
Received preference	322 (23.0)
No preference/indifferent	745 (53.3)
Total	1398 (100)

Patients with missing baseline data (n=6, 0.4%) and missing follow-up scores (n=190, 11.9%) excluded.

After adjustment for severity of condition at baseline (that is, baseline scores), age, and sex, those who did not receive their preference were more likely to return their questionnaire at first follow-up than were those who were indifferent (odds ratio 1.70, 95% confidence interval 1.08 to 2.69; P=0.02); those who did receive their preference also had an increased response rate, but this difference was not significantly different from the indifferent group (odds ratio 1.26, 0.82 to 1.94; P=0.29). The comparison of those who did not get their preference with those who did yielded an odds ratio of 1.35, but this was not significant (95% confidence interval 0.78 to 2.33; P=0.29). The total number of participants included in the analysis to compare the effects of preferences on attrition was 1583 (table 5).

**Table 5 tbl5:** Number (percentage) of participants included in analysis to estimate effect of patients’ preferences on attrition

Preferences	Data at first follow-up		Total
	Data	Missing data	
Did not receive preference	329 (23.6)	27 (14)	356 (22.5)
Received preference	320 (23.0)	34 (18)	354 (22.4)
No preference/indifferent	744 (53.4)	129 (68)	873 (55.1)
Total	1393 (100)	190 (100)	1583 (100)

Patients with missing baseline data (n=6, 0.4%), missing data for sex (4, 0.3%), and missing data for age (1, 0.1%) excluded.

## Discussion

In this review, we identified 17 fully randomised preference trials and obtained patient level data for 11 of these. These trials covered a range of conditions but were mainly in musculoskeletal medicine where patients are unblinded to their treatment and self report quality of life outcomes. In these types of trials, preferences are most likely to have an effect on outcomes and attrition. In contrast with an earlier review,^5^ we have found some evidence to suggest that preferences can modify treatment outcomes, although these effects were not all in the expected direction. In terms of increased study attrition, which is widely hypothesised to be affected by preference, we found the converse of what was expected: participants with a preference who are randomised to the opposite treatment are actually more likely to return follow-up data than those who were indifferent.

### Strengths and limitations

By pooling the data from the eight musculoskeletal trials, we obtained a relatively large sample size (n=1398 to investigate the effect of patients’ preferences on treatment outcomes and n=1583 to investigate the effect of patients’ preferences on attrition). The design of this study—in which we made comparisons between patients who received their preference, patients who did not receive their preference, and those with no preference—is, as far as we can determine, novel for detecting effects of preference.

In most of the trials included in the analysis, the questionnaires had been completed “alone” by the participants and therefore were unlikely to have been influenced by any treatment preference held by health professionals or researchers involved in the trials. Where questionnaires had been completed in the presence of health professionals or researchers, procedures had been put in place by the trialists to minimise the influence of their treatment preferences on the participants’ answers to the questionnaires.

Our study does have some limitations. Firstly, we restricted our meta-analysis to musculoskeletal trials, so our results may not be applicable to other areas of health care. Secondly, within each of the individual component trials participants with a very strong preference for usual care will tend not to be recruited into the studies. However, several studies did recruit participants who had a preference for treatments that were available outside of the trial setting. Thirdly, we interpreted missing data at the first follow-up after the intervention as attrition. We did not investigate if data were present at the final follow-up. Fourthly, eight of the 17 studies were identified through the personal knowledge of one of the authors (DT), who was a co-investigator/author on some reports and who had found others through general reading of trial reports. Although four of these studies were unpublished at the time of the search, this suggests that other similar trials exist that our search strategy did not identify. Finally, confounding could be present—for example, actual treatment associated with preferences causing an effect. We acknowledge that an intermediate confounding variable may be responsible for the association, but we cannot find any evidence of a variable in such an analysis.

### Comparison with other studies

This is the first study that we are aware of in which a patient level meta-analysis has been done to investigate the effects of patients’ preferences on treatment outcomes and attrition. Our review differs from the earlier review by King and colleagues,^5^ in that we sought only trials in which all participants had been randomised irrespective of their preferences. Also our study was restricted to musculoskeletal trials. The search in the review by King and colleagues covered the period 1966 to September 2004 and identified only two fully randomised patient preference trials.^13^ ^w1^ Most of the trials identified in our study were published after this time, indicating that fully randomised patient preference trials are becoming more widely used. By using these designs, we can ensure that when we compare patients who receive their preferred treatment with those who did not receive their preferred treatment they are similar in terms of potential baseline confounders.

### Implications

In terms of treatment effects, we did see an increased treatment effect size among participants who were randomised to their preferred treatment compared with those who were indifferent to the treatment allocation. Preferences seemed to affect outcome despite the fact that patients with and without a preference were balanced in the most important variable at baseline (that is, baseline score). Whether preferences will materially affect the overall outcome of any one trial will depend on the proportion of participants with a preference, the effect of the preference itself, and the effect size of the intervention. We did not look at the origin of preferences, which may involve the person having had that particular treatment before and knowing that the treatment has helped and what the treatment involves. In this review, we have shown that about 57% of the participants have a preference and those who are allocated to their preference show about 0.162 of a standard deviation increase in effect above those who were indifferent. This might have an important effect on the outcomes of a trial. If, for example, a treatment had an effect size of 0.25 among indifferent participants, then our results suggest that for those with a preference the effect would be about 0.41 (that is, a 64% increase in effect size). This increased effect is not necessarily a bias—it might be a genuine effect in the sense that the treatment works better among patients who desire and receive their preferred treatment. It could, however, change the cost effectiveness of a treatment. A treatment may not be cost effective overall but could be among a subgroup of participants who prefer that treatment.^14^ However, we cannot assume this to be true for people with strong preferences for a treatment and who consequently did not take part in the trials included in this study. The behaviour of this group of people is unknown—for example, they may become easily disappointed if the treatment does not meet their expectations. Consequently, we would conclude that in trials—particularly when a novel therapy is available only within the context of the study and outcomes are subjective—pre-randomised preferences should be identified and recorded so that they can be accounted for in any analysis. Although an effect size of 0.162 seems to be modest, we need to set this in the context of generally modest effect sizes from effective treatments for musculoskeletal problems in general. For example, the UK BEAM trial, which evaluated manipulation with or without exercise for the treatment of low back pain, found an effect size of 0.34 for exercise after three months and an effect size of 0.39 for manipulation.^15^ Our results provide no evidence that “resentful demoralisation” leads to a reduction in effect size if people are allocated to their undesired treatment.

Stratifying by preferences would help the power of a trial if either the trial was small or the proportion of patients with a preference was small; if, for instance, 10% of participants had a preference and the trial only had 100 participants, all 10 (or at least eight or nine) participants with a preference might end up in one group. Consequently, we would be unable to control for the possible effects of preference in a regression analysis. Therefore, in small trials or trials in which the proportion of patients with a preference is small, trialists might consider using preference as a stratification variable.

### Conclusions

This review shows that treatment preferences affect outcomes in a sample of musculoskeletal trials but that they are not detrimental to attrition rates. The fully randomised preference trial seems to be more widely used now, which would enable further work to determine the treatment effects of patients’ preferences in different clinical conditions.

**What is already known on this topic**

The effect of patients’ preferences on treatment outcomes in randomised controlled trials is uncertain
Alternative, partially randomised, trials have been designed to overcome the potential problem of patients’ preferences in trials
An existing systematic review found no evidence that preferences influenced attrition and some evidence of an effect on outcomes in partially randomised trials

**What this study adds**

Patients’ preferences do affect treatment outcomes in randomised controlled trials in musculoskeletal medicine
“Resentful demoralisation” did not occur in participants who did not get their preferred treatment
Use of the standard randomised controlled trial, which collects preference data before randomisation, provides the opportunity to take preference effects into account in the analysis

## Notes

This paper was a joint collaboration with the members of the Preference Collaborative Group: Simon J Adamson, J Martin Bland, Elaine M Hay, Ruth E Johnson, Gareth T Jones, Henry Kitchener, Jennifer A Klaber Moffett, Gary J Macfarlane, Hugh MacPherson, Sionnadh McLean, Linsey Nelson, Chris Salisbury, Elaine Thomas, Helen E Tilbrook, and David J Torgerson.

Contributors: JMB provided advice on the statistical analysis and conceived the framework of the statistical analysis. HET did the systematic review as part of her MSc dissertation, did the analysis, and wrote the first draft of the paper. DJT had the idea for the review, supervised HET, identified some papers for inclusion in the review, and helped to write the first draft of the paper. SJA, EMH, REJ, GTJ, HK, JAKM, GJM, HMacP, SMcL, LN, CS, and ET contributed data to the study. SJA, JMB, EMH, GTJ, JAKM, GJM, HMacP, LN, CS, ET, and DJT critically reviewed versions of the manuscript. HET is the guarantor.

Provenance and peer review: Not commissioned; externally peer reviewed.

## Notes

**Cite this as: ***BMJ* 2008;337:a1864

## References

[1] Pocock SJ. Clinical trials: a practical approach. Chichester: John Wiley and Sons, 1983.

[2] Brewin CR, Bradley C. Patient preferences and randomised clinical trials. BMJ 1989;299:313-5. 2504416 10.1136/bmj.299.6694.313 PMC1837157

[3] McPherson K, Britton AR, Wennberg JE. Are randomised trials controlled? Patient preferences and unblind trials. J Roy Soc Med 1997;90:652-6. 9496288 10.1177/014107689709001205 PMC1296732

[4] Howard L, Thornicroft G. Patient preference randomised controlled trials in mental health research. Br J Psychiatry 2006;188:303-4. 16582054 10.1192/bjp.188.4.303

[5] King M, Nazareth I, Lampe F, Bower P, Chandler M, Morou M, et al. Impact of participants and physician intervention preferences on randomised trials: a systematic review. JAMA 2005;293:1089-99. 15741531 10.1001/jama.293.9.1089

[6] Torgerson DJ, Klaber-Moffett J, Russell IT. Patient preferences in randomised trials: threat or opportunity? J Health Serv Res Policy 1996;1:194-7. 10180870 10.1177/135581969600100403

[7] McPherson K, Britton AR, Wennberg JE. Are randomised trials controlled? Patient preferences and unblind trials. J Roy Soc Med 1997;90:652-6. 9496288 10.1177/014107689709001205 PMC1296732

[8] McPherson K, Chalmers I. Incorporating patient preferences into clinical trials [letter]. BMJ 1998;317:78. PMC1113468 9651286

[9] Silverman WA, Altman DG. Patients’ preferences and randomised trials. Lancet 1996;347:171-4. 8544555 10.1016/s0140-6736(96)90347-5

[10] Halpern SD. Evaluating preference effects in partially unblinded, randomized clinical trials. J Clin Epidemiol 2003;56:109-15. 12654404 10.1016/s0895-4356(02)00598-x

[11] Torgerson D, Sibbald B. Understanding controlled trials: what is a patient preference trial? BMJ 1998;316:360. 9487173 10.1136/bmj.316.7128.360 PMC2665528

[12] King M, Nazareth I, Lampe F, Bower P, Chandler M, Morou M, et al. Conceptual framework and systematic review of the effects of participants’ and professionals’ preferences in randomised controlled trials. Health Technol Assess 2005;9(35):1-186, iii-iv. 10.3310/hta9350 16153352

[13] Hardy GE, Barkham M, Shapiro D, Reynolds S, Rees A. Credibility and outcome of cognitive-behavioural and psychodynamic-interpersonal psychotherapy. Br J Clin Psychol 1995;34:555-69. 8563662 10.1111/j.2044-8260.1995.tb01489.x

[14] Manca A, Epstein DM, Torgerson DJ, Klaber-Moffett JA, Coulton S, Farrin AJ, et al. Randomized trial of a brief physiotherapy intervention compared with usual physiotherapy for neck pain patients: cost-effectiveness analysis. Int J Technol Assess Health Care 2006;22:67-75. 16673682 10.1017/s0266462306050859

[15] UK BEAM Trial Team. United Kingdom back pain exercise and manipulation (UKBEAM) randomised trial: effectiveness of physical treatments for back pain in primary care. BMJ 2004;329:1377, doi:10.1136/bmj.38282.669225.AE 15556955 10.1136/bmj.38282.669225.AE PMC535454

[16] Shapiro DA, Barkham M, Rees A, Hardy GE, Reynolds S, Startup M. Effects of treatment duration and severity of depression on the effectiveness of cognitive-behavioural and psychodynamic-interpersonal psychotherapy. J Consult Clin Psychol 1994;62:522-34. 8063978 10.1037/0022-006x.62.3.522

[17] Kendrick T, Simons L, Mynors-Wallis L, Gray A, Lathlean J, Pickering R, et al. A trial of problem-solving by community mental health nurses for anxiety, depression and life difficulties among general practice patients: the CPN-GP study. Health Technol Assess 2005;9(37):1-104, iii. 10.3310/hta9370 16153354

[18] Pepper J, Lamb S, Doughty G, Fereday Smith J. Female urinary incontinence: women’s preferences for group or individual treatment. BMC Women’s Health (in press).

[19] Sherman KJ, Cherkin DC, Erro J, Miglioretti DL, Deyo RA. Comparing yoga, exercise, and a self-care book for chronic low back pain: a randomized, controlled trial. Ann Intern Med 2005;143:849-56. 16365466 10.7326/0003-4819-143-12-200512200-00003

[20] Simpson S, Corney R, Fitzgerald P, Beecham J. A randomized controlled trial to evaluate the effectiveness and cost-effectiveness of counselling patients with chronic depression. Health Technol Assess 2000;4(36):1-83. 11134918

[21] Unützer J, Katon W, Callahan CM, Williams JW, Hunkeler E, Harpole, L, et al. Collaborative care management of late-life depression in the primary care setting: a randomized controlled trial. JAMA 2002;288:2836-45. 12472325 10.1001/jama.288.22.2836

